# Supplementary material for: Drastic increase in the magnitude of very rare summer-mean vapor pressure deficit extremes
Source: Nat Commun. 2024 Aug 15;15:7022. doi: 10.1038/s41467-024-51305-w (PMC11327300; doi:10.1038/s41467-024-51305-w)
Supplement: Supplementary file 1 — Supplementary Information [file 41467_2024_51305_MOESM1_ESM.pdf]

Drastic increase in the magnitude of very rare summer-mean vapor pressure deficit extremes: Supplementary Information

Including:

- Supplementary Method 1
- Supplementary Figures 1–8

Supplementary Method 1

In the following, we detail the derivation of the process attribution to the ΔI of $\mathrm{VPD_{S+}}$ between the historical and end-of-century period. In particular, we focus on refining ΔI_T, starting with Eq. 14 and arriving at Eq. 15 shown in the methods (both in the main text):

$$\Delta I = \langle I \rangle^{eoc} - \langle I \rangle^{hist} = \langle I_T \rangle^{eoc} - \langle I_T \rangle^{hist} + \Delta I_{qT} + \Delta I_{qd} + \Delta res \tag{1}$$

where angle brackets denote the average over all $\mathrm{VPD_{S+}}$ in one hemisphere and in the respective period, which is denoted by the superscript (note that these brackets are omitted in the main text whenever possible without loss of clarity).

For $\langle I_T \rangle^{hist}$ and $\langle I_T \rangle^{eoc}$ we proceed as illustrated in the following at the example of $\langle I_T \rangle^{eoc}$. We insert the average over all considered $\mathrm{VPD_{S+}}$ of the corresponding term in Eq. 13 (in the main text). Then, we re-write the resulting term as products of means. Hereby it is important to note that $\langle a \cdot b \rangle \neq \langle a \rangle \cdot \langle b \rangle$ but rather $\langle a \cdot b \rangle = \langle a \rangle \cdot \langle b \rangle + cov(a,b)$. Hence $\langle I_T \rangle^{eoc}$ becomes:

$$\langle I_T \rangle^{eoc} = \left\langle \left[T' \cdot \left. \frac{\partial \mathrm{VPD}}{\partial T} \right|_c \right] \right\rangle^{eoc} = \langle [T'] \rangle^{eoc} \cdot \left\langle \left[\left. \frac{\partial \mathrm{VPD}}{\partial T} \right|_c \right] \right\rangle^{eoc} + res \tag{2}$$

where the residual summarizes covariance effects. The square brackets denote the average across all land grid cells of one $\mathrm{VPD_{S+}}$, and angle brackets with superscript *eoc* then denote averages over the considered $\mathrm{VPD_{S+}}$ in the 2091–2100 period. Next, we express only $\langle I_T \rangle^{eoc}$, expanded according to Supplementary Eq. 2, in terms of the respective historical terms and the respective deltas. That is, all mean values in the end-of-century period are rewritten as the sum of their mean value in the historical period plus a change in the mean value, i.e., $\langle [a] \rangle^{eoc} = \langle [a] \rangle^{hist} + \Delta a$. Thus,

$$\langle I_T \rangle^{eoc} - \langle I_T \rangle^{hist} = \left(\langle [T'] \rangle^{hist} + \Delta T' \right) \cdot \left(\left\langle \left[\left. \frac{\partial \mathrm{VPD}}{\partial T} \right|_c \right] \right\rangle^{hist} + \Delta \left. \frac{\partial \mathrm{VPD}}{\partial T} \right|_c \right) - \langle [T'] \rangle^{hist} \cdot \left\langle \left[\left. \frac{\partial \mathrm{VPD}}{\partial T} \right|_c \right] \right\rangle^{hist} + res \tag{3}$$

where the residual again summarizes the effects of covariance (changes). Supplementary Equation 1 now reads:

$$\Delta I = \underbrace{\Delta T' \cdot \left\langle \left[\left. \frac{\partial \mathrm{VPD}}{\partial T} \right|_c \right] \right\rangle^{hist}}_{\mathcal{T}_{\mathrm{var}}} + \underbrace{\Delta \left. \frac{\partial \mathrm{VPD}}{\partial T} \right|_c \cdot \langle [T'] \rangle^{hist}}_{\mathcal{T}_{\mathrm{clim}}} + \underbrace{\Delta I_{qT} + \Delta I_{qd}}_{\mathcal{Q}} + \mathcal{E} = \mathcal{T}_{\mathrm{var}} + \mathcal{T}_{\mathrm{clim}} + \mathcal{Q} + \mathcal{E} \tag{4}$$

This equation includes all so-called processes that contribute to ΔI, namely the change in I that would result from changing T' only (i.e., in absence of a mean warming; $\mathcal{T}_{\mathrm{var}}$), changes in I that would result from a mean warming, i.e., increasing T_c, but constant T' ($\mathcal{T}_{\mathrm{clim}}$), the effect of changing q-contributions to I ($\mathcal{Q}$), and a residual $\mathcal{E}$. The $\mathcal{E}$ summarizes several terms related to covariance and higher orders, which, as we will show a posteriori, are comparably small.

Supplementary Figures

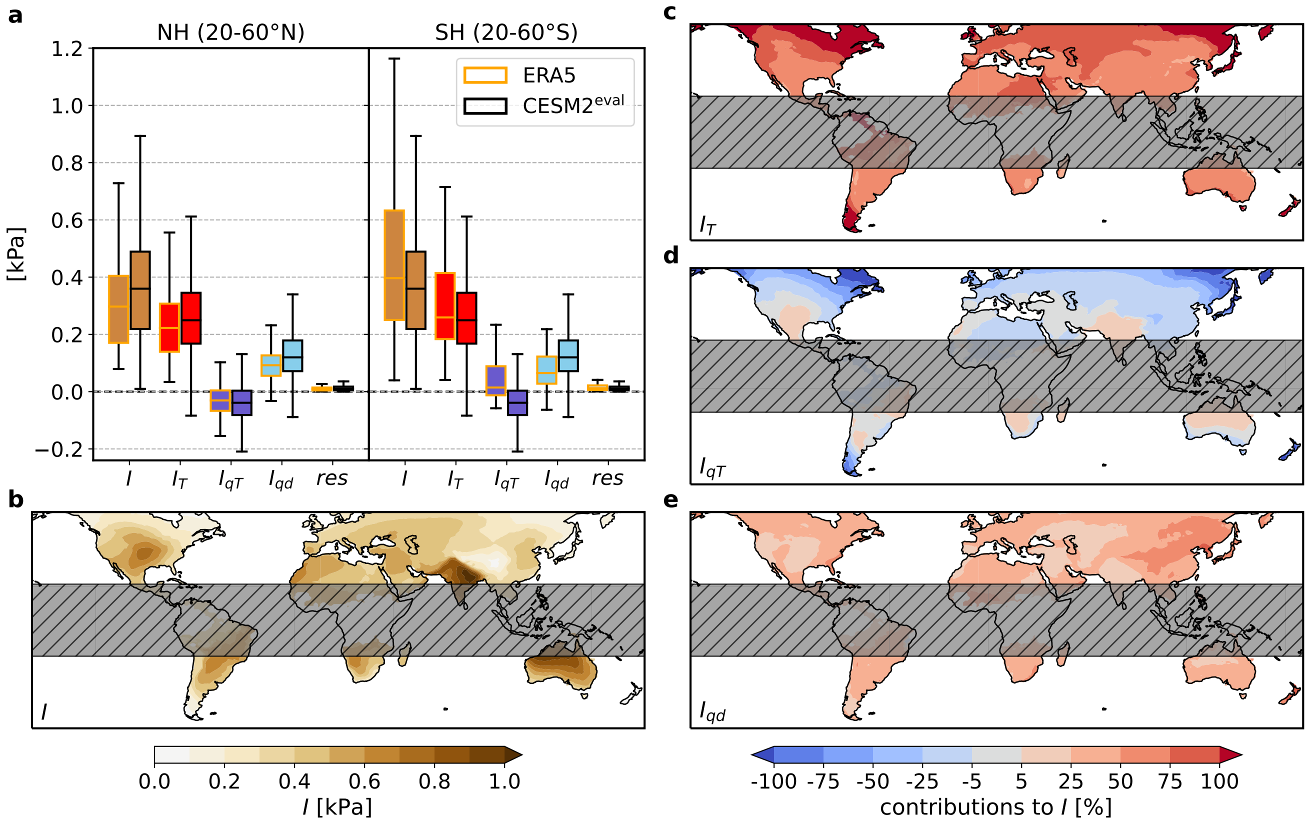

Supplementary Figure 1. Additional information regarding the results displayed in Fig. 2. **a**, Variability in intensity (I) and its contributions for summer vapor pressure deficit extremes (VPD_{S+}) in ERA5 and CESM2^{eval} shown as box-plot including median line, a box from the first to the third quartile (IQR), and whiskers extending to the farthest data point lying within 1.5 times the IQR. **b**, The same as Fig. 2b. **c–e**, Relative contributions to I from temperature anomalies (I_T) (**c**), from the climatological co-variability between temperature and specific humidity (I_{qT}) (**d**), and from dynamically induced humidity anomalies (I_{qd}) (**e**).

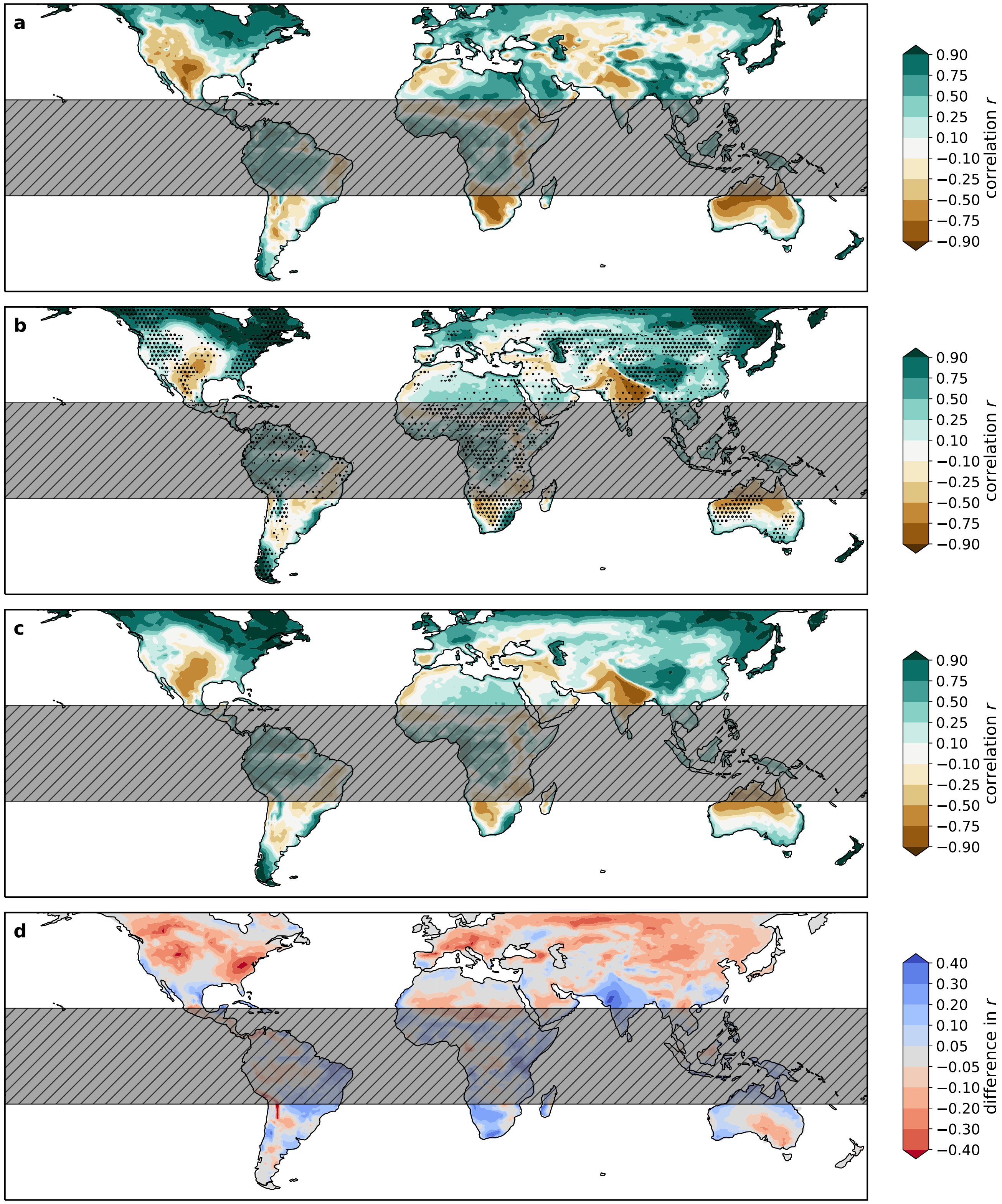

Supplementary Figure 2. Correlation between seasonal temperature (T) and humidity (q) and its change between the hist and eoc period. **a-c**, The Pearson correlation coefficient r between non-detrended q and T in 1979–2023 in ERA5 (**a**), and averaged over all CESM2^{eval} (**b**) and CESM2^{hist} subsets (**c**). Dense and sparse stippling in **b** indicate regions where the ERA5 value lies below and above the range spanned up by the CESM2^{eval} subsets, respectively. **d**, Difference in r averaged over subsets in CESM2^{eoc} minus that in CESM2^{hist}. Regions with grey hatching are not considered in this study.

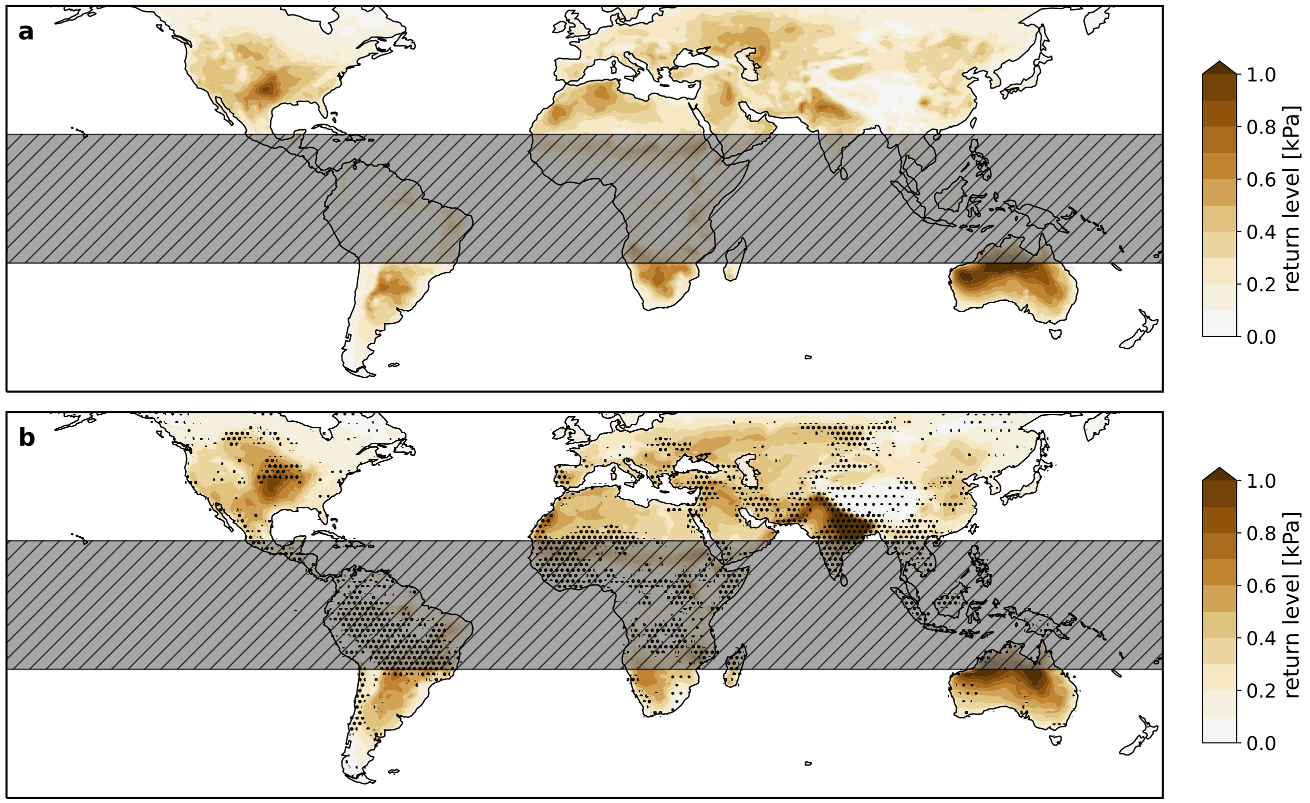

Supplementary Figure 3. The 40-year return level of the vapor pressure deficit anomaly (VPD'). **a**, The 40-year return level of summer VPD' in ERA5, which can be inferred directly from the fitted Yeo-Johnson transformed normal distribution at each grid point (see Methods). **b**, The average 40-year return level of summer VPD' over all subsets in CESM2^{eval}. Dense and sparse stippling in **b** indicate regions where the ERA5 value lies below and above the range spanned up by the CESM2^{eval} subsets, respectively.

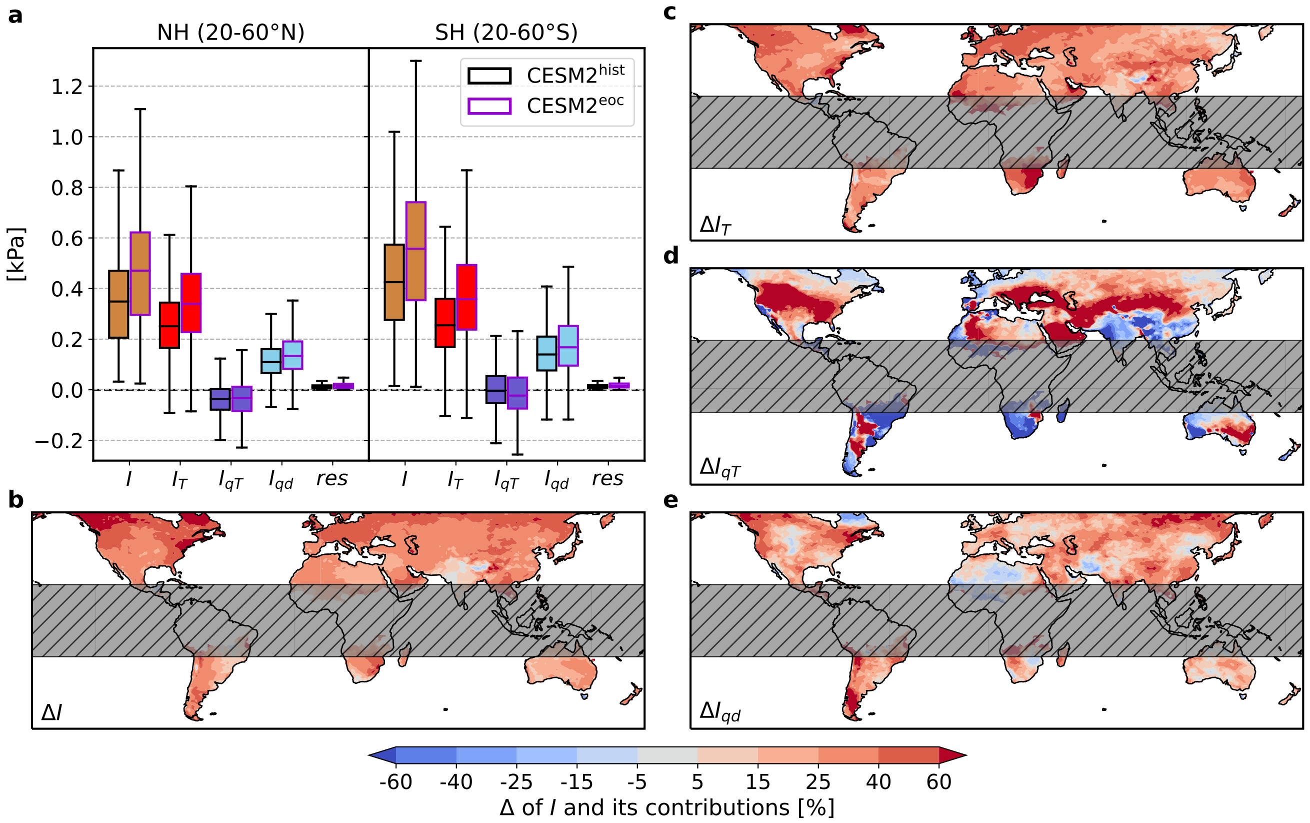

Supplementary Figure 4. Additional information regarding the results displayed in Fig. 3. **a**, Variability in intensity (I) and its contributions for summer vapor pressure deficit extremes ($\mathrm{VPD}_{\mathrm{S+}}$) in CESM2^{hist} and CESM2^{eoc} shown as box-plot (analogous to box-plot shown in Supplementary Fig. 1). **b-e**, The relative change from the hist to eoc period of I (**b**), of contributions from temperature anomalies (I_T) (**c**), of contributions from the climatological co-variability between temperature and specific humidity (I_{qT}) (**d**), and of contributions from dynamically induced humidity anomalies (I_{qd}) (**e**), computed as the respective Δ divided by the magnitude (in absolute terms) of the respective value in CESM2^{hist}.

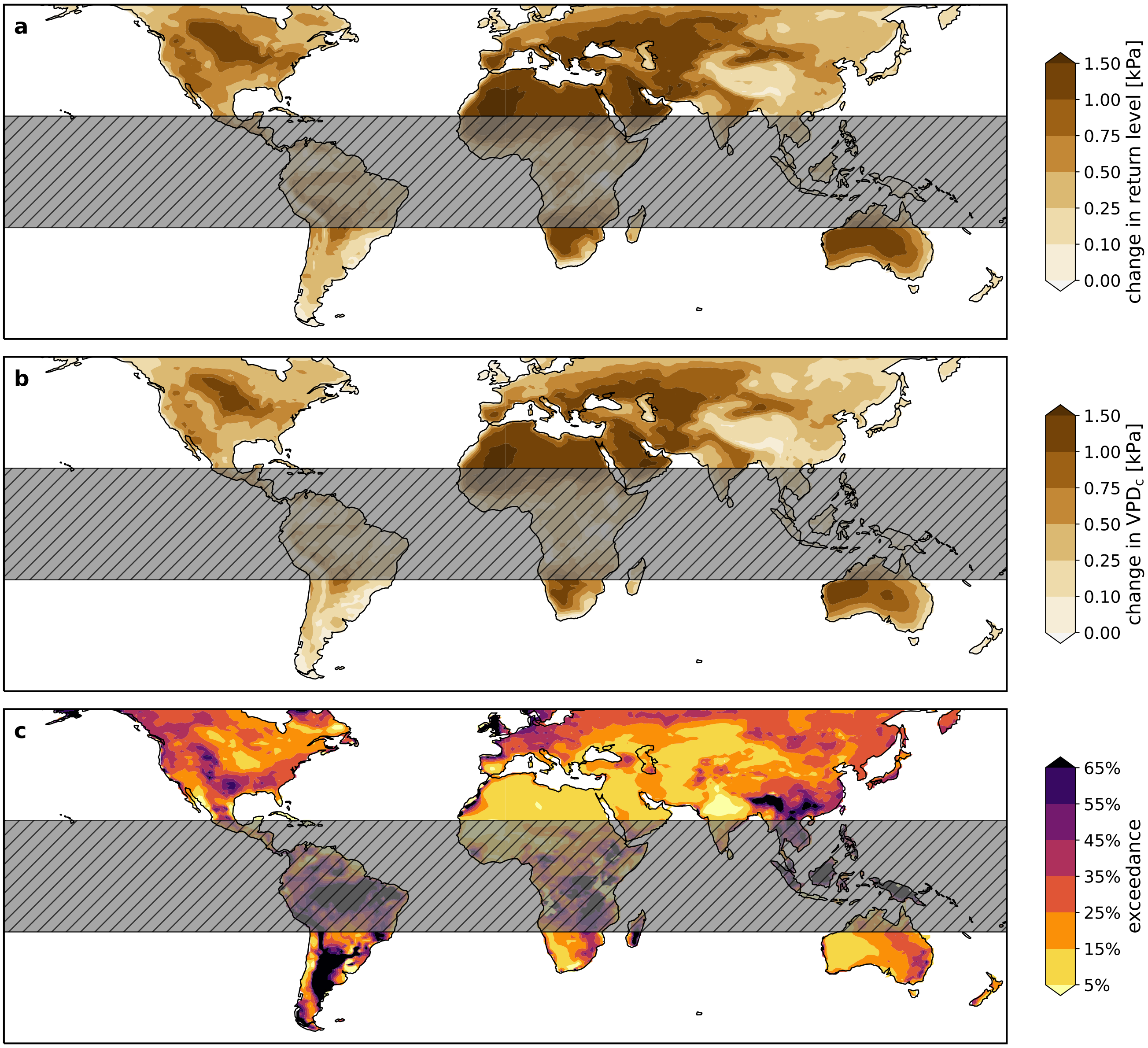

Supplementary Figure 5. Difference between $CESM2^{eoc}$ and $CESM2^{hist}$ (eoc−hist) of the absolute 40-year return level, i.e., of the sum of the 40-year return level of the vapor pressure deficit anomaly (VPD'; as shown in Supplementary Fig. 3) and the climatological mean VPD (VPD_c), compared to differences in VPD_c only. **a,b**, Differences in the absolute 40-year return level (**a**) and in VPD_c (**b**). **c**, The percentage by which values shown in **a** exceed those shown in **b**. Note that in **c**, some extremely positive and negatives values occur where changes in VPD_c are marginal.

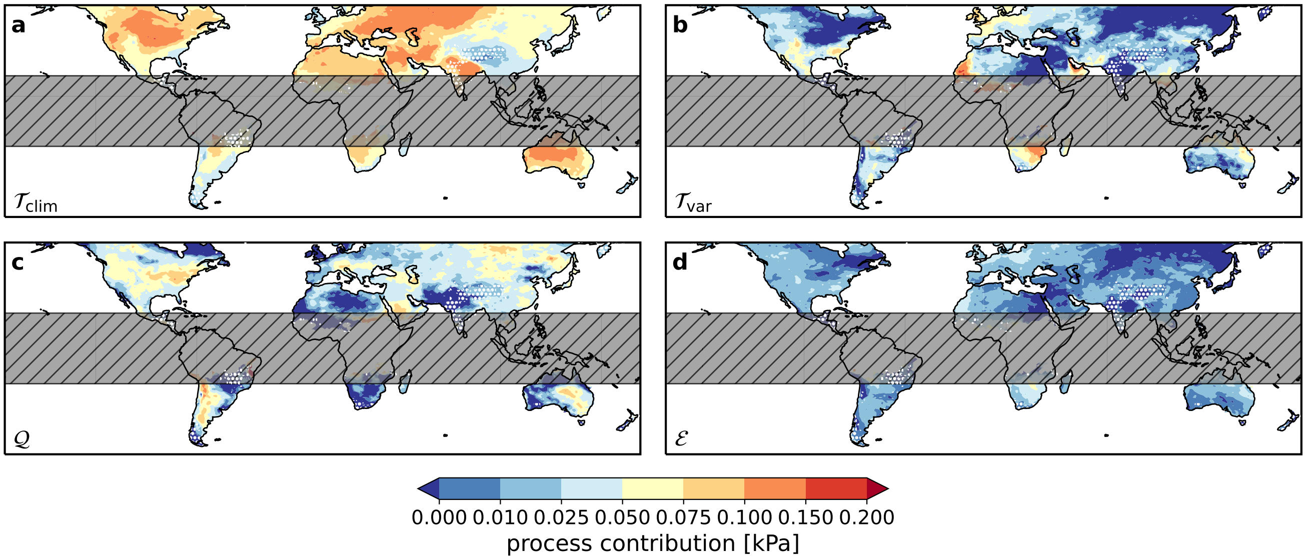

Supplementary Figure 6. Spatial representation of the processes contributing to the intensification (ΔI) of $\text{VPD}_{\text{S+}}$ between the hist and eoc periods. Absolute contributions (i.e., in units kPa) to ΔI from $\mathcal{T}_{\text{clim}}$ (**a**), $\mathcal{T}_{\text{var}}$ (**b**), $\mathcal{Q}$ (**c**), and $\mathcal{E}$ (**d**), whereby values at every grid cell are calculated over all $\text{VPD}_{\text{S+}}$ that comprise the respective grid cell. White stippling denotes regions where $\Delta I < 0.05$ kPa. Regions with grey hatching are not considered in this study.

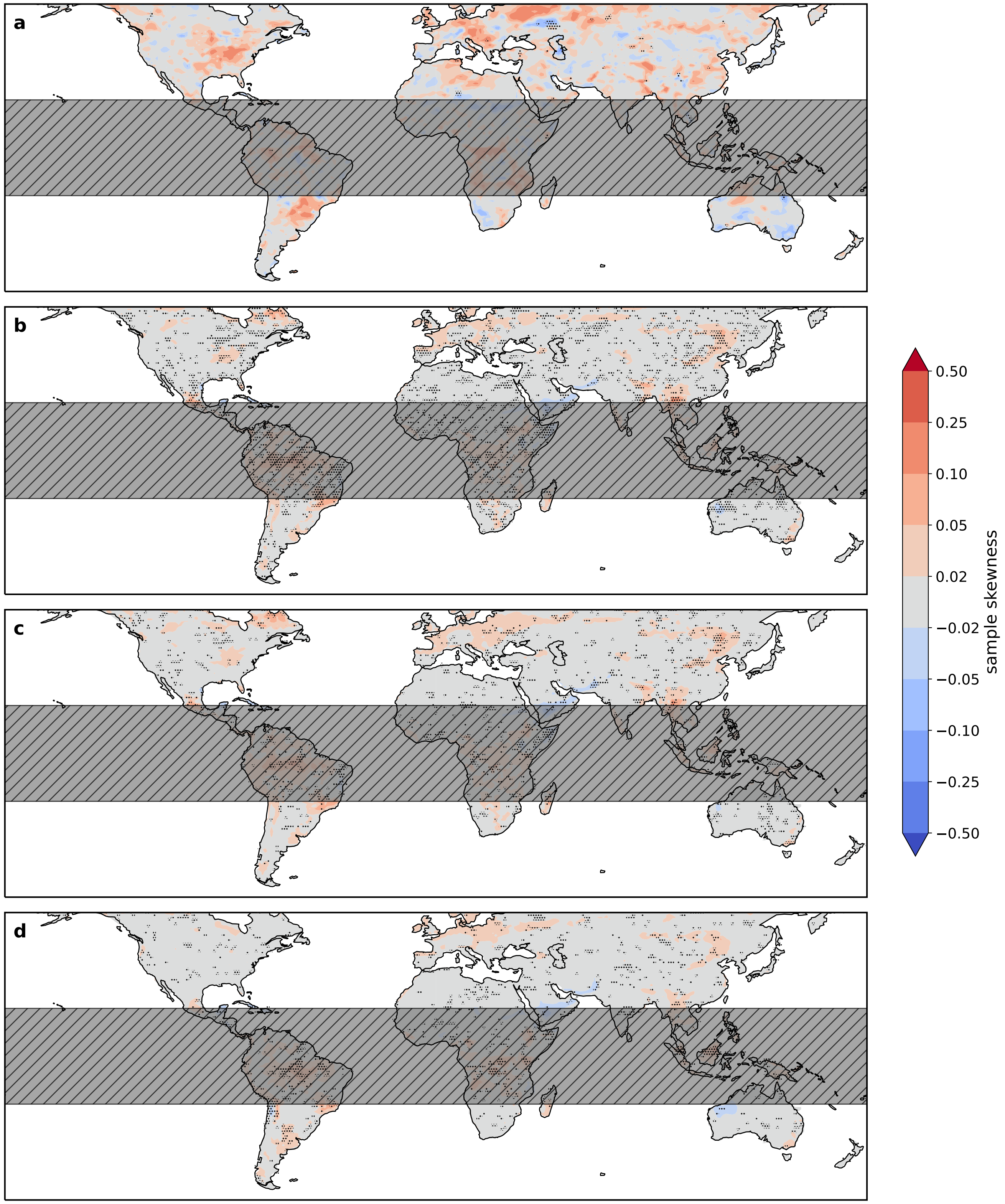

Supplementary Figure 7. Sample skewness of the transformed vapor pressure deficit anomaly (VPD') in ERA5 (**a**), CESM2^{eval} (**b**), CESM2^{hist} (**c**), and CESM^{eoc} (**d**). Values in **b-d** refer to the mean skewness of all CESM2 subsets in the respective dataset. Stippling in **a** indicates grid cells where the ERA5 distribution of transformed VPD' deviates from the normal distribution according to a Shapiro-Wilks test (α = 5%). Stippling in **b-d** indicates where this is the case for more than one CESM2 subset in the respective dataset. Regions with grey hatching are not considered in this study.

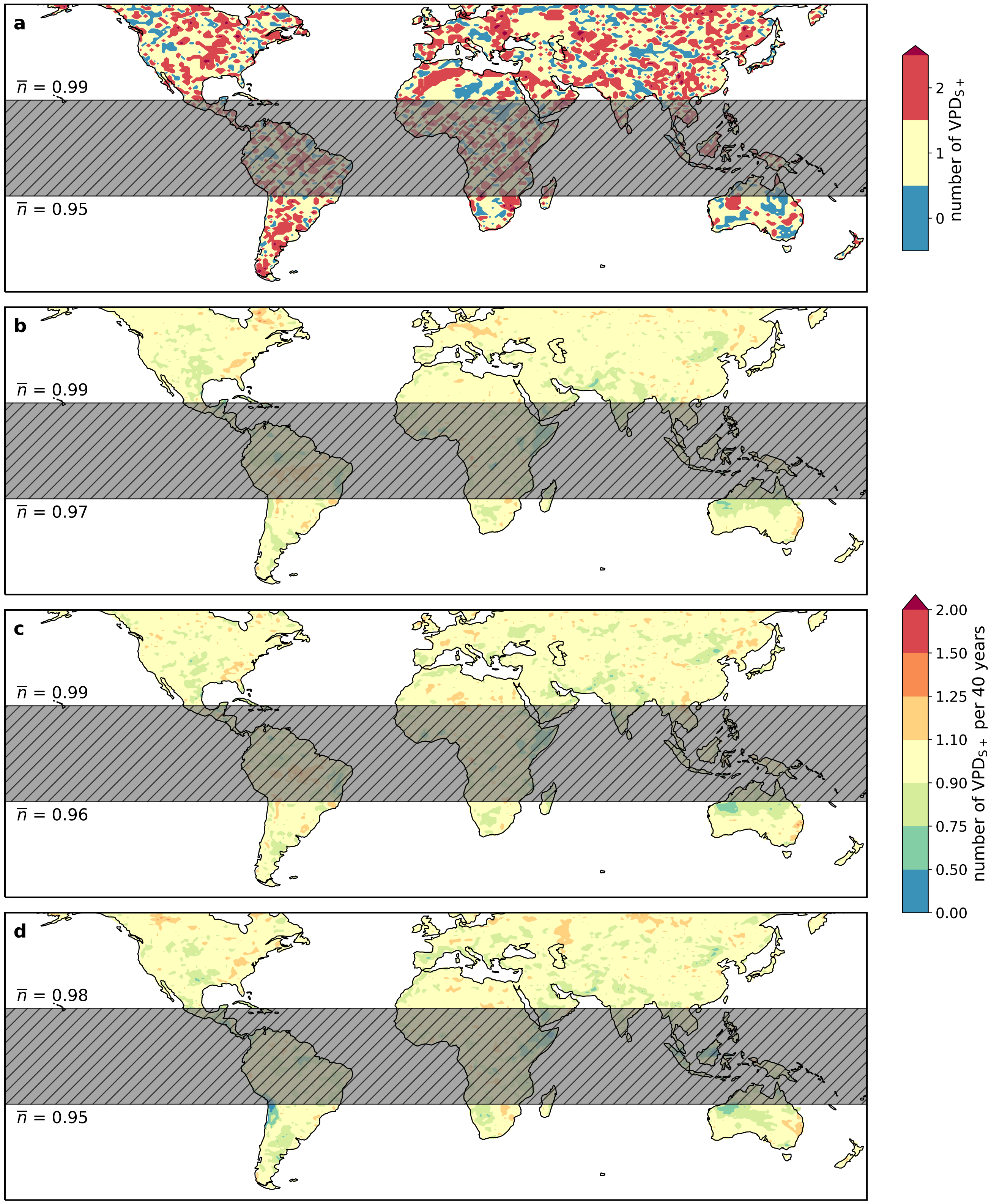

Supplementary Figure 8. The number of summer vapor pressure deficit extremes (VPD_{S+}; not yet filtered according to their minimum land area or location of occurrence) per grid cell. **a**, The total number of VPD_{S+} identified in ERA5 (45 years in 1979–2023). **b-d**, The number of VPD_{S+} identified per 40 years in $CESM2^{eval}$ (**b**), $CESM2^{hist}$ (**c**), and $CESM2^{eoc}$ (**d**). Inset numbers denote the land average number of VPD_{S+} per 40 years for all datasets across both mid-latitudes. Regions with grey hatching are not considered in this study.
